# Interoperabilität im Gesundheitswesen: auch für digitale Gesundheitsanwendungen (DiGA) verordnet

**DOI:** 10.1007/s00103-021-03414-w

**Published:** 2021-09-16

**Authors:** Stefanie Weber, Kai U. Heitmann

**Affiliations:** 1 Abteilung „Kodiersysteme“, Bundesinstitut für Arzneimittel und Medizinprodukte, Kurt-Georg-Kiesinger-Allee 3, 53175 Bonn, Deutschland; 2 health innovation hub, Bundesministerium für Gesundheit, Berlin, Deutschland

**Keywords:** Interoperabilität, DiGA, Digitalisierung, Standardisierung, Elektronische Patientenakte, Interoperability, DiGA, Digitalization, Standardization, Electronic health record

## Abstract

Digitale Gesundheitsanwendungen (DiGA) sind eines der Räder im Getriebe des digitalen Gesundheitswesens. Wie alle anderen kommunizierenden Anwendungen müssen DiGA interoperabel sein, damit das ganze System reibungslos funktioniert. Dabei muss Interoperabilität auf 4 verschiedenen Ebenen gegeben sein, dies sind: funktionale und fachinhaltliche Anforderungen; strukturelle und semantische Anforderungen; Anforderungen an Sicherheit und Transport und organisatorische Anforderungen.

In Deutschland wurde in den letzten Jahren ein großer Sprung in ein digitales Gesundheitswesen initiiert, verstärkt durch die Erfahrungen aus der COVID-19-Pandemie. Aktuelle Gesetzgebungen zielen auf eine Festlegung von Standards und einheitlichen Abläufen und etablieren damit den benötigten verbindlichen Rahmen für ein Gesamtkonzept in der Digitalisierung. Interoperable DiGA können mit den anderen Systemen im Gesundheitswesen kommunizieren, wenn es die PatientInnen wünschen. Möglich sind z. B. der Anschluss an die elektronische Patientenakte (ePA) und eine damit einhergehende Datenspende für Forschungszwecke. So können PatientInnen nicht nur direkt von dem positiven Versorgungseffekt einer DiGA profitieren, sondern auch indirekt durch die Datenspende zur Forschung und damit zur Verbesserung des Gesundheitswesens beitragen.

## Abstract (en)

Digital health applications (DiGA) are a cog in the machine of a digital health system that must be interoperable like all other communicating applications in order to function smoothly. Interoperability takes place at four levels: functional subject definition of content, semantic and syntactic standardization, security and transport requirements, and organizational aspects.

In Germany, a major leap towards a more digital healthcare system has been initiated in recent years, reinforced by the experience gained from the COVID-19 pandemic. Current legislation aims at a uniform definition of standards and processes and thus establishes the required binding framework for an overall concept in digitization. DiGA can communicate with other healthcare systems using the same semantic and syntactic standardizations if the patient so desires. With the possible connection to electronic patient records and the accompanying data donation option, patients can benefit more than once through interoperable DiGA – not only through the direct positive care effect of the digital health application, but also indirectly through data donation, which can contribute to improving the entire healthcare system through appropriate research.

## Interoperabilität definiert

Im 21. Jahrhundert ist die Digitalisierung der Treiber für Veränderungen. Prozesse und Kommunikation ändern sich hierbei grundlegend und werden durch Digitalisierung kürzer und schneller. Auch im Gesundheitswesen ist die Digitalisierung schon lange angekommen und verändert immer mehr Behandlungen und Abläufe. Durch die COVID-19-Pandemie ist allen Beteiligten bewusst geworden, welche Vorteile eine Digitalisierung des Gesundheitswesens haben kann. Dies hat eine neue Offenheit für die Möglichkeiten der Digitalisierung mit sich gebracht.

Auch in der Gesetzgebung im Gesundheitswesen ist in den letzten Jahren ein deutlicher Schub in Richtung digitaler Gesundheitsversorgung erfolgt. Neben den „digitalen Gesundheitsanwendungen“ (DiGA) sind Gesetzesinitiativen bspw. zur elektronischen Patientenakte (ePA) und damit einhergehenden Regelungen zu Datenfluss, Datenschutz und Interoperabilität maßgeblich.

Ein wichtiges Schlagwort bei der Digitalisierung ist „Interoperabilität“. Doch was genau ist das überhaupt? Der Duden definiert Interoperabilität als „Fähigkeit unterschiedlicher Systeme, möglichst nahtlos zusammenzuarbeiten“ [1]. Eine möglichst nahtlose digitale Zusammenarbeit kann zwischen 2 Systemen erreicht werden, indem die Übermittlung von Informationen strukturell und inhaltlich standardisiert wird: die sogenannte syntaktische und semantische Standardisierung.

Im Gesundheitssystem gibt es nun aber nicht nur 2, sondern viele Systeme, die für eine vollständige Digitalisierung interoperabel gemacht werden müssen, und hierzu gehören auch neuere Anwendungen, wie die DiGA. Damit in allen Anwendungen die gleichen semantischen und syntaktischen Standards verwendet werden, hat der Gesetzgeber einige Regelungen getroffen, die die verschiedenen Ebenen der Interoperabilität adressieren und die notwendige Verbindlichkeit schaffen.

## Ebenen der Interoperabilität

Interoperabilität ist demnach die Fähigkeit der nahtlosen Zusammenarbeit zwischen Systemen, Organisationen oder Techniken. Vor allem zielt diese Fähigkeit aber darauf ab, dass Menschen kollaborieren. Interoperabilität ist deshalb in erster Linie ein soziales Konzept: Menschen müssen miteinander diskutieren, gemeinsame Festlegungen treffen, gemeinsam lernen, sich vertrauen, um dann Daten interoperabel austauschen zu können. Auf diesen Aspekt kommen wir noch zurück.

Aus mehr technischer Sicht ist Interoperabilität die Fähigkeit von 2 oder mehr Systemen, Informationen

- auszutauschen,
- diese zu verstehen und
- wiederzuverwenden.

**Figure 1:**
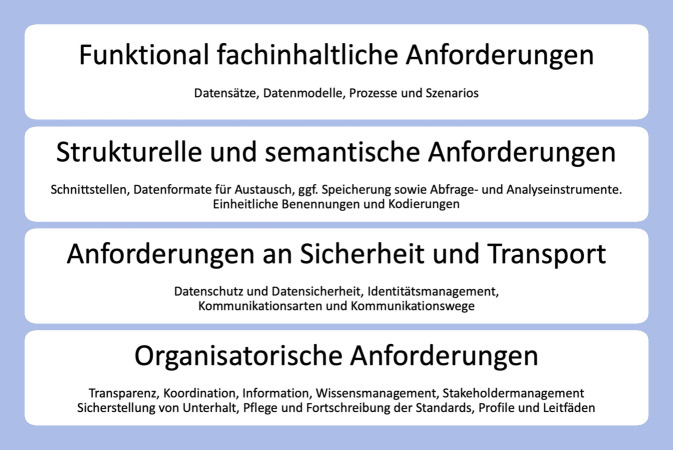

Um diese Aufgaben zu bewerkstelligen, wird Interoperabilität häufig in Ebenen von Anforderungen (Abb. 1) gesehen, die im Folgenden näher beleuchtet werden.

### Funktional fachinhaltliche Anforderungen

An erster Stelle steht bei der Erlangung von Vereinbarungen zur Interoperabilität die fachinhaltliche – also zum Beispiel medizinische/pflegerische – Dokumentation der Anforderungen für den Austausch von Informationen. Dabei beschreibt man, welche Datenelemente wie und in welcher Häufigkeit aus welchem Anlass erhoben werden sollen. Dies mündet typischerweise in sogenannten Datensätzen und Informationsmodellen sowie Prozessbeschreibungen.

Datensätze wie auch Informationsmodelle beschreiben genau, um welche Daten es sich handelt und wie diese miteinander zusammenhängen. Prozessbeschreibungen belegen, wann welche Daten zwischen den im Kommunikationsgeschehen eingebundenen Akteuren ausgetauscht werden. So legt man beispielsweise fest, dass für die Dokumentation einer Coronainfektion, etwa durch eine App, den PatientInnen eine Liste von möglichen Symptomen angeboten wird, aus der ausgewählt werden kann und dass bei Fieber die Körpertemperatur als Messparameter in Grad Celsius angegeben wird.

Die fachinhaltlichen Vorgaben werden auf der nächsten Ebene aufgenommen und weiterbearbeitet.

### Syntaktische und semantische Anforderungen

Als Empfänger Informationen zu verstehen und sie wiederzuverwenden, ist Gegenstand der *semantischen Interoperabilität*: ein gemeinsames und gleiches Verständnis über Begrifflichkeiten. Das erreicht man in vielen Fällen nur durch genaue Beschreibung der Konzepte und die Verbindung mit einem „Code“, denn Sprache ist vielfältig oder allein nicht immer eindeutig.

Auch wenn Menschen beispielsweise die Worte „renale Insuffizienz“ und „Niereninsuffizienz“ als gleich interpretieren können, so sind das für die Kommunikation zwischen 2 IT-Systemen unterschiedliche Dinge. Versteht das sendende IT-System nur das Wort „renale Insuffizienz“, weil es seine Programmierung so vorgesehen hat, und beim empfangenden System ist für diese medizinische Diagnose das Wort „Niereninsuffizienz“ hinterlegt, so kann das jeweils andere System den Inhalt der Nachricht nicht „verstehen“. Die beiden Systeme sind nicht semantisch interoperabel. Hat das empfangende System nun beispielsweise eine Warnmeldung für bestimmte Medikamente vorgesehen, die bei Niereninsuffizienz nicht verwendet werden sollen, kann es bei Empfang des Wortes „renale Insuffizienz“ nicht reagieren, ein Nachteil für die Patientensicherheit.

Ein einheitlicher Code, wie beispielsweise der ICD-10-GM-Code „N19“ [3], hilft für die digitale Kommunikation weiter, doch handelt es sich bei Codes aus dieser Klassifikation oft um Krankheitsgruppen, die mehrere Diagnosen in Klassen zusammenfassen. Für eine feiner granulierte semantische Standardisierung bedarf es deshalb einer medizinischen Terminologie wie SNOMED CT [4], mit der sich sehr weite Bereiche der Kommunikation im Gesundheitswesen semantisch standardisieren lassen.

Nicht nur verschiedene Begriffe für denselben Sachverhalt sind ein Problem, auch gleiche Begriffe mit verschiedenen Bedeutungen.

**Figure 2:**
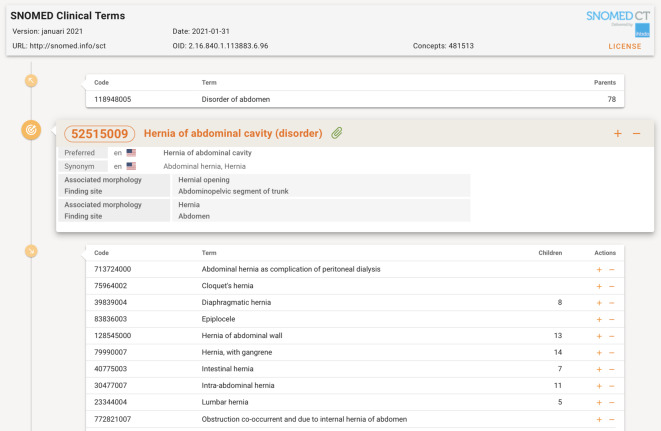

Am Begriff „Bruch“, der in der Medizin nur im Kontext korrekt verstanden werden kann, wird dies deutlich. Handelt es sich um einen Bauchdeckenbruch (Hernie) oder um einen Knochenbruch (Fraktur)? Synonyme, genauere Beschreibungen und ein eindeutiger Code, beispielsweise aus dem Codesystem SNOMED CT, machen Kommunikation sicher und unmissverständlich. Konkret im Beispiel ist die Hernie (als Gesundheitsstörung) eindeutig mit „SNOMED 52515009“ gekennzeichnet (Abb. 2), die Fraktur hätte den abweichenden Code 125605004.

Auf diese Weise werden textliche Informationen mit zusätzlichen Codes „angereichert“, annotiert, somit eindeutig und für Computeranwendungen nutzbar. Mit einem „Netzwerk“ von Codes, wie es die SNOMED-Terminologie bietet, können innovative Softwareapplikationen sichere und optimale Dokumentation durch den menschlichen Benutzer fördern.

Die Ebene der syntaktischen, also der *technischen oder strukturellen Interoperabilität,* bezeichnet die Fähigkeit, Informationen von einem Ort zum anderen zu bringen und Regeln zur Übertragung (Formate und sicheres Senden z. B. über ein Netzwerk) zu definieren. Auch die Vereinbarung einer bestimmten Reihenfolge oder Hierarchie von Information sowie deren Eigenschaften (Typ) gehören dazu. Für einen Personennamen wird für die interoperable Übertragung festgelegt, dass zunächst der Familienname übertragen wird, gefolgt von den Namensteilen, die der Unterscheidung der Familienmitglieder dienen, im Deutschen meist Vornamen genannt.

Auch die Angabe eines Datums, ein einfacher Datentyp, erfolgt nach interoperablen Vorgaben der International Standardization Organization (ISO), wie bspw. im Standard ISO 8601: z. B. „2021-04-08“. Hier wird aber auch deutlich, dass das Übertragungsformat der Daten zwar interoperabel sein muss, die Darstellung für das menschliche Gegenüber aber in „gewohnter“ Manier („8. April 2021“) erfolgen kann.

### Anforderungen an Sicherheit und Transport sowie organisatorische Anforderungen

In unmittelbarer Nähe zur strukturellen Interoperabilität sind die Anforderungen an Sicherheit und Transport angesiedelt. Austausch und Zugriff auf Daten darf nur unter dafür vorgesehenen gesetzlichen Rahmenbedingen geschehen. Das hat auch Auswirkungen auf organisatorische Anforderungen, also wie und in welchem Rahmen Organisationen sich vertrauen und zusammenarbeiten.

In Deutschland werden die technischen Erfordernisse des Austauschs von Informationen im Gesundheitswesen im Wesentlichen über die sogenannte Telematikinfrastruktur (TI) und die dafür entsprechend festgelegten Spezifikationen geregelt. Darin werden Zugriffsmöglichkeiten, Datensicherheits- und Datenschutzaspekte definiert, nach denen sich alle Kommunikationsteilnehmende richten müssen. Auf der TI sind Dienste zur Kommunikation verankert, wie etwa KIM (Kommunikation im Medizinwesen) als sicheren „E-Mail“-Dienst unter vertrauenswürdigen Partnern, der Abgleich von Versicherteninformationen oder die bereits erwähnte elektronische Patientenakte.

## Interoperabilität in der Versorgung am Beispiel der DiGA

Für die DiGA hat der Gesetzgeber bereits von Beginn an Interoperabilität „verordnet“. So heißt es in der Digitale-Gesundheitsanwendungen-Verordnung (DiGAV; [5]):

§ 5 Anforderungen an Qualität

(1) Digitale Gesundheitsanwendungen sind so zu gestalten, dass sie die Anforderungen der technischen und semantischen Interoperabilität umsetzen. Insbesondere muss die digitale Gesundheitsanwendung ermöglichen, dass von der digitalen Gesundheitsanwendung verarbeitete Daten in geeigneten interoperablen Formaten exportiert und im Rahmen der Versorgung genutzt werden können. Zudem muss die digitale Gesundheitsanwendung interoperable Schnittstellen verwenden, wenn es im Rahmen der bestimmungsgemäßen Nutzung der digitalen Gesundheitsanwendung vorgesehen ist, dass die digitale Gesundheitsanwendung Daten mit vom Versicherten genutzten Medizingeräten oder mit vom Versicherten getragenen Sensoren zur Messung und Übertragung von Vitalwerten (Wearables) austauscht.

**Figure 3:**
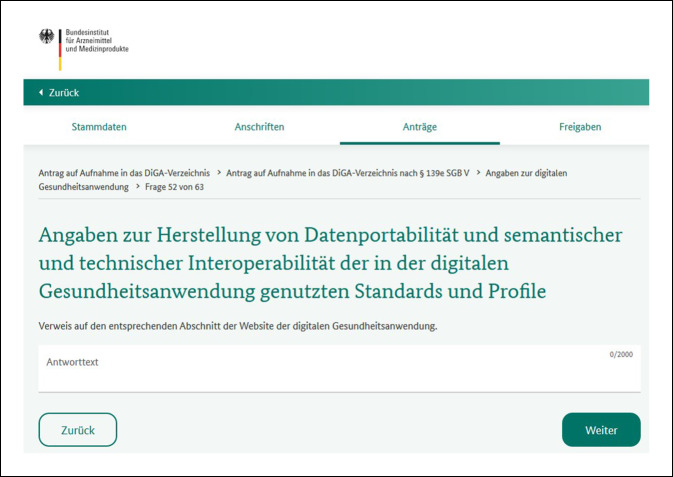

Bei der Interoperabilität im Gesundheitswesen ist die Bestrebung der Bundesregierung auch eine Zusammenführung der Patientendaten in der elektronischen Patientenakte (ePA), die es den PatientInnen ermöglichen soll, souverän auf ihre Gesundheitsdaten zuzugreifen und diese schnell und tagesaktuell beispielsweise einer behandelnden Person zugänglich zu machen. Auch Daten, die PatientInnen mit ihren DiGA sammeln, sollen auf Wunsch der PatientInnen in die ePA fließen können. Zumindest müssen sie aber in standardisierter Form auch dem behandelnden medizinischen Personal zugänglich gemacht werden können, sodass diese z. B. die Funktion und Wirkung der DiGA für die jeweils behandelte Person beurteilen und ggf. eine Fortführung oder Beendigung der Therapie mit der DiGA veranlassen können. Hierfür wird der Aspekt der Interoperabilität der Daten auch bei dem Antrag auf Aufnahme ins DiGA-Verzeichnis explizit abgefragt (Abb. 3).

Die Inhalte der ePA werden jedoch nicht ausschließlich aus DiGA-Daten gespeist. Es gibt noch viele weitere Informationsobjekte, die in der ePA abgelegt werden können: Labordaten, Impfungen, Medikation, Arztbriefe, weitere Informationen von Behandelnden oder Einrichtungen, sogar der Bericht eines Notfalleinsatzes im Ausland ist denkbar. Zwar steht Deutschland hier in der Umsetzung der gesetzlichen Vorgaben noch am Anfang, doch hat die aktuelle Gesetzgebung die Weichen nun auf eine schnelle Digitalisierung umgelegt.

**Figure 4:**
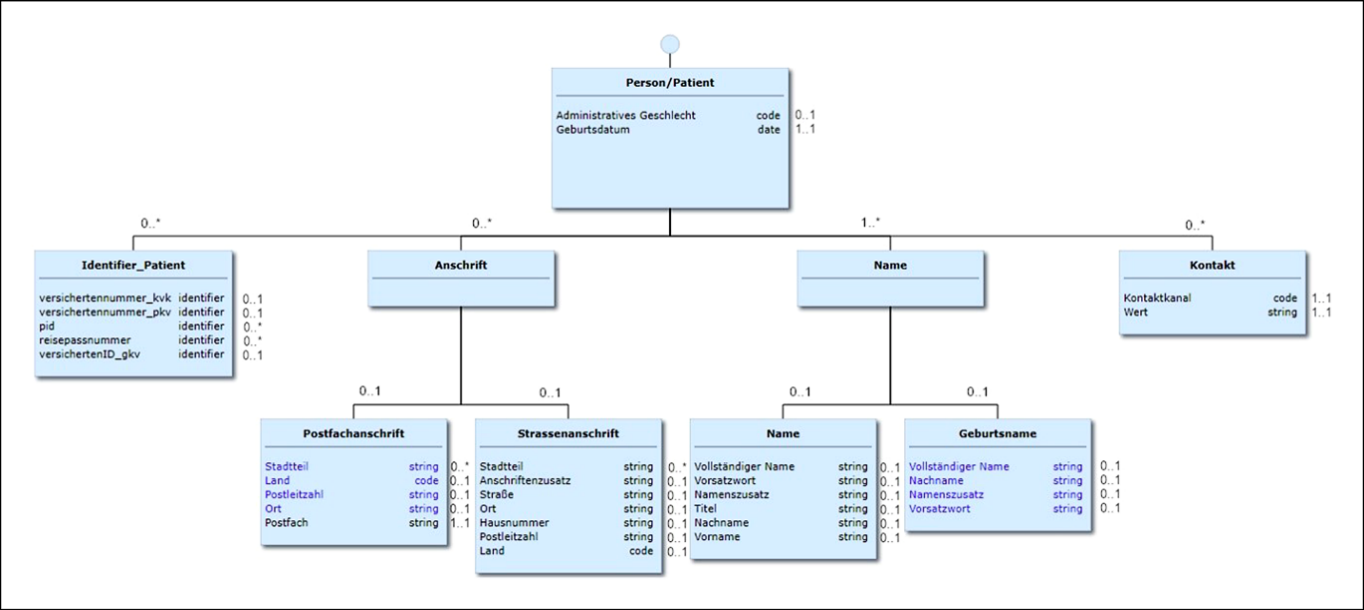

Die Kassenärztlichen Bundesvereinigung (KBV) legt die Inhalte der ePA semantisch und syntaktisch standardisiert fest, wodurch gewährleistet werden soll, dass alle Informationen, die in die ePA einfließen, einem diesbezüglichen Gesamtkonzept unterliegen. Hierbei kommen internationale Standards wie HL7 FHIR zur Anwendung. Die KBV stimmt die Festlegungen in einem breit angelegten Prozess mit allen Beteiligten ab und stellt sie dann in Form von „Medizinischen Informationsobjekten (MIO)“ zur Anwendung bereit [7]. Durch Basisprofile werden immer wiederkehrende Teilaspekte der MIO definiert, wie beispielsweise die Daten zur Patientenidentifikation (Abb. 4 und 5).

**Figure 5:**
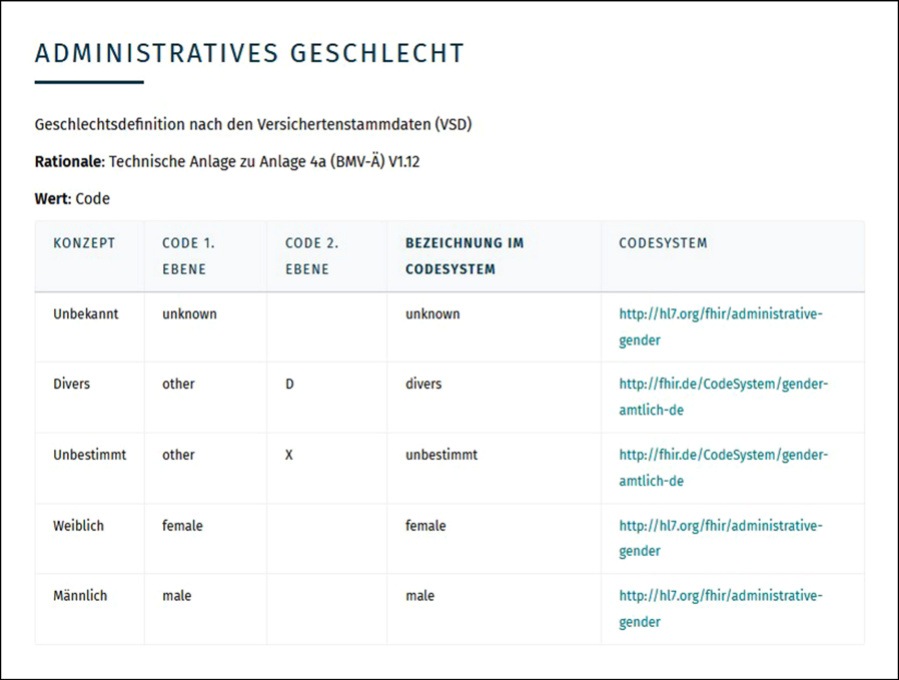

In den DiGA sollen diese Festlegungen der MIO nun ebenfalls zur Anwendung kommen und müssen von den Herstellern bei der Programmierung entsprechend berücksichtigt werden. Hierdurch wird gewährleistet, dass beim Austausch von Daten Informationen von den empfangenden Systemen semantisch und syntaktisch „verstanden“ werden.

Möchten die PatientInnen nun ihre Daten auch noch für die Forschung spenden: Das Patientendaten-Schutz-Gesetz hat eine entsprechende Regelung im fünften Sozialgesetzbuch (SGB V) etabliert:

§ 363 Verarbeitung von Daten der elektronischen Patientenakte zu Forschungszwecken

(1) Versicherte können die Daten ihrer elektronischen Patientenakte freiwillig für die in § 303e Absatz 2 Nummer 2, 4, 5 und 7 aufgeführten Forschungszwecke freigeben.

Um die Daten auswerten zu können, bedarf es ebenfalls einer einheitlichen Datenstandardisierung, semantisch wie auch syntaktisch. Und hier schließt sich der Kreis auch für die PatientInnen, die auf mehreren Ebenen von ihren eigenen Daten u. a. aus den DiGA profitieren können:

- Durch Interoperabilität mit den Systemen ihrer Behandelnden wird beispielsweise auf Interaktionen zwischen verschiedenen Behandlungen (z. B. Medikamenten) hingewiesen und die behandelnde Person kann die Behandlung bestmöglich anpassen.
- Durch Interoperabilität mit den ePA werden die Daten der PatientInnen aus mehreren Behandlungssettings auswertbar, beispielsweise ein Blutzuckerverlauf aus einem DiGA-Diabetes-Tagebuch und aus verschiedenen anderen Quellen und Zeitpunkten, was zu einer präziseren Behandlung führen kann.
- Durch Datenspende nach SGB V §363 Abs. 1 wird auf einer breiten standardisierten Forschungsdatenbasis eine deutlich verbesserte Auswertung von Daten zu einer Verbesserung der medizinischen Behandlung beitragen können, von der letztendlich dann auch die Person profitieren kann, die ihre DiGA-Daten gespendet hat.

Im Bereich der Forschung hat sich in den letzten Jahren die sogenannte Medizininformatik-Initiative (MII), ein vom Bundesministerium für Bildung und Forschung finanziertes Projekt, unter anderem zur Aufgabe gemacht, auch hier interoperable Strukturen und Terminologien zu definieren und nutzbar zu machen.

Eingangs wurde betont, dass vor allem die soziale Komponente eine wichtige Rolle bei der Interoperabilität spielt. Am Beispiel der MIO der KBV und der Definitionen im Rahmen der MII ist dies gut zu sehen: Wo immer möglich fußen die Vorgaben auf denselben Definitionen, um so größtmögliche Zusammenarbeit zu gewährleisten. Dies gilt auch für weitere vom Gesetzgeber initiierte Unternehmungen wie das Bestätigungsverfahren für Informationssysteme im Krankenhaus (ISIK), für das die gematik GmbH verantwortlich ist [10]. Die verschiedenen Gruppen haben sich hier intensiv zuvor über die Rahmenbedingen und Vorgaben (sogenannte Profile) ausgetauscht, sodass ein größtmögliches Maß an Abstimmung erreicht wurde.

Letztlich fußen alle diese Profile auf den deutschen Basisprofilen, die im nationalen Interoperabilitätsforum und entsprechenden Arbeitsgruppen gemeinsam erarbeitet worden sind. MIO und die Profile der MII verfeinern diese Basisprofile für die jeweiligen Anwendungsfälle. Das Interoperabilitätsforum ist Teil der „Interoperabilitäts-Community“ in Deutschland, in dem ExpertInnen für jeden Rede und Antwort stehen, der sich mit der praktischen Anwendung von Standards beschäftigt, Fragen hat und sich weiterbilden möchte. So lernt man hier voneinander und schafft interoperable Lösungen.

## Interoperabilität kennt keine Landesgrenzen?

Im 21. Jahrhundert leben Menschen nicht nur in einem deutlich digitaler werdenden Umfeld, sie sind auch deutlich mobiler. Wie sieht es also mit der grenzübergreifenden Interoperabilität von Gesundheitsdaten aus?

Auch hier hat der Gesetzgeber entsprechende Orientierung in den Regelungen zur ePA vorgesehen, die auch in der DiGAV [6] verankert sind. Bei der Auswahl von semantischen und syntaktischen Standardisierungen soll primär auf international etablierte Standards zurückgegriffen werden. In § 355 SGB V wird in Absatz 6 geregelt, dass die KBV „bei ihren Festlegungen nach Absatz 1 grundsätzlich internationale Standards zu nutzen“ hat.

Reist eine Person nun in ein anderes Land, so sollte im Fall einer dort notwendigen medizinischen Behandlung diese idealerweise basierend auf den für die Person vorhandenen medizinischen Informationen erfolgen. Hierfür ist im Vorfeld die Zustimmung der Person erforderlich. In der Europäischen Union (EU) gibt es bereits seit mehreren Jahren eine Richtlinie über die Ausübung der Patientenrechte in der grenzüberschreitenden Gesundheitsversorgung [11]. Im Kapitel IV der Richtlinie wird in Artikel 14 der Rahmen für die Zusammenarbeit der Länder in der EU zum Thema Interoperabilität von Gesundheitsdaten gesetzt. Aber auch darüber hinaus ist auf europäischer Ebene in den letzten Jahren eine Zunahme der Bestrebung zur Herstellung von Interoperabilität zu Gesundheitsdaten festzustellen. Unter dem Schlagwort „My Health@EU“ soll die Vernetzung der elektronischen Gesundheitsdienste zwischen den Ländern ausgebaut werden [12]. Auch hierfür werden Anstrengungen zur semantischen und syntaktischen Standardisierung der Daten vorangetrieben und sollen mit den deutschen Festlegungen bestmöglich übereinstimmen. Primär werden Daten zur Patientenkurzakte und zu elektronischen Verschreibungen adressiert. Doch werden in großen geförderten Projekten auch europäische Datenforschungspools geplant, so beispielsweise im gerade gestarteten Projekt „TEHDAS“ [13]. Zielsetzung ist auch für die EU die „Vernetzung und der Austausch von Gesundheitsdaten für Forschung, schnellere Diagnose und bessere Gesundheit“, wie in einer Mitteilung der Kommission aus 2018 dargelegt wird [14].

Letztendlich ist es also nötig, dass eine DiGA in ein Standardisierungsumfeld eingepasst wird, das sowohl die Anforderungen an semantische und syntaktische Standardisierung für Deutschland als auch darüber hinaus berücksichtigt.

## Fazit und Ausblick

Wir haben in Deutschland erst jüngst damit begonnen, konkrete Vorgaben zur Interoperabilität gesetzlich zu verankern und damit die nötige Verbindlichkeit zu schaffen. Während andere Länder wie Österreich oder die Schweiz hier einige Jahre Vorsprung haben, holt Deutschland auf und kann sich moderner und innovativer Methoden und Standards bedienen. Noch sind wir am Anfang, Impfpass und Mutterpass waren erste Inhaltsdefinitionen für die ePA, es folgen aber bald Dokumentationen für die Pflege, Laborberichte, Entlassberichte und vieles mehr. Bemerkenswert ist allemal, dass sich hier vor allem eine „Community“ herausgebildet hat, die die Grundlage für einen regen und zielgerichteten, vertrauenswürdigen interoperablen Austausch darstellt – zwischen Menschen und mittels Computer.

Die DiGA sind ein weiteres Rad, das das komplexe Getriebe der Digitalisierung des Gesundheitswesens vorantreibt. Um auch bei den DiGA keine „Sollbruchstellen“ zu etablieren, ist es essenziell, dass diese die gleichen interoperablen Standards wie andere Anwendungen verwenden. Hierfür müssen sich die Hersteller der DiGA mit den festgelegten Standards auseinandersetzen und diese für die gesetzlich festgelegte Möglichkeit der Überführung der Daten in die ePA berücksichtigen [15]. In einem iterativen Prozess werden deshalb die semantischen und syntaktischen Standardisierungen durch die KBV kontinuierlich erweitert werden.

Letztendlich zählt für den Anwendenden einer DiGA, dass ihm auch durch diesen Baustein seiner Behandlung ein positiver Effekt für seine Gesamtbehandlung zuteilwird. Durch die Interoperabilität der jeweiligen DiGA mit anderen Anwendungen und insbesondere mit der ePA und der damit einhergehenden möglichen Datenspende für Forschungszwecke kann die anwendende Person nachhaltiger und umfassender von der DiGA profitieren.
